# Effect of task interruption on the situation awareness of air traffic controllers

**DOI:** 10.1371/journal.pone.0314183

**Published:** 2024-11-22

**Authors:** Kun Zhou, Chenglong Wang, Siqi Xie, Yanan Zhou, Xuelong Zhang, Yanqing Wang, Hui Tang

**Affiliations:** 1 College of Safety Science and Engineering, Civil Aviation University of China, Tianjin, People’s Republic of China; 2 College of Foreign Languages, Civil Aviation University of China, Tianjin, People’s Republic of China; 3 School of Vocational Education, Tianjin University of Technology and Education, Tianjin, People’s Republic of China; Catholic University of the Sacred Heart: Universita Cattolica del Sacro Cuore, ITALY

## Abstract

Multiple intersecting situational tasks in the field of aviation often cause air traffic controllers to face issues pertaining to interruption and task switching. To investigate the impact of task interruption on the situation awareness of air traffic controllers, two experiments were conducted. Experiment 1, which focused on 44 new graduates preparing to work in the field of air traffic control, revealed that task interruption reduced the participants’ levels of situation awareness. Experiment 2, which focused on 80 new air traffic control graduates, employed a 2 (primary task modality: visual, auditory) × 2 (interruption modality: visual, auditory) between-subjects design and revealed that the negative effect of task interruption on situation awareness was moderated by task modality. Interruptions that occur in the same modality as the primary task were shown to result in greater decreases than were cross modal interruptions. Visual interruption led to a greater decrease in situation awareness than did auditory interruption, and interruption of the visual task also caused a greater decrease in situation awareness than did interruption of the auditory task. These findings might be valuable in attempts to enhance situation awareness among air traffic controllers by providing insights into the design of human‒computer interactions in the context of an air traffic control automation system.

## Introduction

Situation awareness refers to the understanding needed to operate a complex system in a rapidly changing task environment [1,2]. As situation awareness is presumed to facilitate the timely, efficient, and safe movement of air traffic in response to changes in the system state, this factor is perhaps the most critical for air traffic controllers, who play a critical role in managing the safe and orderly flow of air traffic [3]. Controllers who exhibit poor situation awareness make more technical errors (such as height readback errors) and cognitive errors (such as perceptual errors and memory errors), which might entail disastrous consequences [4–6]. In a study of accidents among major airlines, 88% of such accidents involving human error could be attributed to problems with situation awareness [7–9].

Given the key role played by situation awareness problems in human error, strategies aimed at helping controllers achieve a high level of situation awareness are particularly desirable. Researchers have investigated individual operators and how they acquire situation awareness cognitively during task performance [10,11]. Such researchers have proposed that individuals’ ability to obtain and maintain situation awareness in a complex, dynamic task environment exhibits tremendous variations. Therefore, it is possible to improve situation awareness at the individual level through targeted training or selection processes (such as cognitive ability testing, experience evaluation, and situation awareness measurement) [12,13]. Underlying this approach is the theoretical premise that the individual’s ability to attain and maintain situation awareness exhibits cross-situational consistency and stability. However, the question of whether an individual who can maintain good situation awareness in one situation can also maintain good situation awareness stably in another situation remains open. Therefore, reliable improvements in situation awareness may rely largely on the optimization of the design of the air traffic control system, especially in terms of the human‒computer interface [14–16]. For example, airports that feature high levels of traffic volume are typically characterized by multiple controller positions. However, this approach does not completely mitigate the negative impacts of task interruptions, as such interruptions are typically universal, inevitable, and immediate components of the concurrent multitasking activities of air traffic control [17,18].

A task interruption usually refers to an event that breaks the continuity of a primary task and causes an attention switch due to the requirement of an immediate response [19]. From an integrated perspective, researchers have divided task interruptions into (a) intrusions, (b) distractions, (c) breaks, (d) surprises, and (e) multitasking [20]. Typical air traffic control operations are characterized by complex information, urgency, and highly dynamic situations [21,22]. To make accurate decisions and take appropriate actions, controllers must quickly understand the state of the rapidly changing system and environment [3,23,24]. In this process, controllers are often required to scan the radar screen and the flight process strip constantly with the goals of identifying the state of the aircraft, contacting pilots via air-to-ground communication, deconflicting air traffic activities, answering calls related to follow-up and coordination, etc. [1]. This variety of tasks featuring highly dynamic contextual complexities inevitably causes frequent task interruptions for controllers that require them to shift their attention quickly among different interfaces and tasks. For example, a controller may identify an impending aircraft conflict (i.e., future violations of the principle of minimum aircraft separation) but be unable to resolve the conflict immediately because of other air traffic [25,26]. During this retention interval, in which the conflict cannot be resolved, the controller is likely to be interrupted by other ongoing task demands, such as air-to-ground communication [27].

A large body of research has investigated the cognitive and emotional challenges and benefits associated with frequent task interruption alongside their impacts on individual-level outcomes [20]. However, their results of such research have been somewhat inconsistent, and the perspectives adopted by previous researchers have rarely been integrated across different areas of interest [28]. Some research has shown that interruptions during the process of task execution are generally disruptive, although the corresponding loss of efficiency has been reported in a variety of ways, such as increased task performance time, decreased accuracy, greater stress, and increased mental workload [29–33]. Other researchers have noted that interruptions may actually be beneficial with regard to performance in certain contexts [34–38]. For example, Zickerick and colleagues reported that they did not find external interference to affect performance under working memory load. Unexpectedly, performance exhibited significant improvements in trials conducted after distractions in comparison with those conducted before distractions [39]. Leroy et al. highlighted the difficulty of comparing, contrasting, and integrating the findings of numerous studies due to the diverse ways in which the term "interruption" has been used to describe different types of task transitions [20].

In addition, previous studies have investigated the effects of task interruptions under sequential task conditions, but few studies have focused on interruptions in a concurrent multitasking environment [18]. Notably, unlike simple sequential tasks, concurrent multitasking involves subtasks pertaining to multiple attributes and entails greater cognitive challenges for operators [40]. For air traffic controllers who work in such a multitasking environment, it is crucial to prioritize the most urgent subtask (i.e., resolving the most urgent aircraft conflicts) after an interruption rather than simply resuming the previously interrupted subtask. This process requires them to maintain a heightened level of situation awareness within a dynamically changing and complex system, including to ensure a safe level of minimum separation between aircraft. Unfortunately, data from incident reports, controller interviews, and laboratory simulations pertaining to air traffic control have revealed that controllers occasionally forget to complete deferred task actions [27,41]. In a study that investigated how the presence and type of interruptions affect the likelihood of individuals remembering to perform deferred tasks in simulated air traffic control as well as the speed with which they perform those tasks, researchers also reported that individuals are particularly susceptible to interference-based forgetting [32]. It can thus be inferred that task interruption has a negative effect on controllers’ ability to maintain situation awareness. In this paper, we report the results of experimental research that involved simulating air traffic control tasks to test this hypothesis.

Interruptions in air traffic control operations may occur in different modalities, including the visual and auditory modalities. Specifically, controllers may be interrupted by urgent requests from pilots during checking the flight process strip. In this study, we are specifically interested in how the modality of an interruption impacts air traffic controllers’ situation awareness. Is one modality less disruptive than another? Are intramodal interruptions more disruptive than cross-modal interruptions? The majority of empirical papers on the impact of the interruption modality on primary task resumption have been based on multiple resource theory, which highlights the advantages of cross-modal information presentation [42]. According to multiple resource theory [43,44], the brain employs multiple resources to process information and can support more efficient multitasking when different resources are utilized. Therefore, all else being equal, cross-modal interruptions (e.g., auditory interruption–visual primary task) are expected to be less disruptive than are interruptions that occur within the same modality as the primary task (e.g., visual interruption–visual primary task). The second purpose of this study is to investigate whether cross-modal interruptions have a less disruptive impact on controllers’ situation awareness than do intramodal interruptions.

Given that air traffic control operations primarily involve visual tasks, the visuospatial subsystem plays a crucial role in the continuous updating of situation awareness [45]. Therefore, it is reasonable to assume that engaging in a secondary visual task is more likely to overwhelm the cognitive processing capabilities of the controller due to an increase in competition for attention resources. Consequently, in comparison with other forms of interruptions (such as auditory interruption–visual primary task, visual interruption–auditory primary task, or auditory interruption–auditory primary task), a visual interruption to a visual primary task could pose a greater threat to situation awareness.

To test these hypotheses, we conducted the present study, which involved two laboratory experiments that aimed to investigate the impact of interruptions on controllers’ situation awareness and to determine whether the modality of the primary/interruption task can moderate this effect.

## Experiment 1

The purpose of this experiment was to investigate how task interruptions affect situation awareness on the part of air traffic controllers. For this purpose, participants were required to perform a simulated air traffic control task as the primary task either with or without an interrupting task.

### Participants

Notably, a significant portion of frontline staff involved in air traffic control are young controllers. These individuals may be more susceptible to decreased situation awareness in response to task interruptions than are their more experienced counterparts due to the limited work experience of the former group. Thus, it is crucial to investigate the impact of task interruptions on the situation awareness of young controllers with the goal of enhancing air traffic safety. Therefore, a total of 52 healthy male volunteers whose average age was twenty-three years were recruited to participate in this experiment. The entry criteria included familiarity with radar control operations; normal visual acuity, hearing, and color vision; and no history of psychiatric or neurological disorders. All the participants were new graduates who had been studying air traffic management for four years and who had completed a radar internship course on the simulation software used in the present study.

Specifically, in order to ensure validity for subsequent data analysis, we utilized G*Power (3.19.7) software to conduct a priori sample size estimation, in which context the statistical power was set at 0.8, the effect size at 0.8, and the alpha value at 0.05. The analysis revealed that a minimum of 52 participants was required to detect a significant effect with 80% power. Initially, we estimated a sample loss of less than 1%. However, due to conflicting schedules and busy graduation-related matters, eight participants withdrew from the experiment prior to its commencement, resulting in a final attrition rate of 1.5% for both groups. Ultimately, only 44 volunteers completed the experiment. Before the experiment started, these participants were advised of the anonymity of their results. Oral informed consent was obtained from each participant. After the participants completed the experiment, they were thanked and debriefed. This study was reviewed and approved by the Research Ethics Committee of the College of Safety Science and Engineering, Civil Aviation University of China.

### Task and materials

The primary task employed in this experiment was a simulated air traffic control task in which participants were asked to use a mouse to click on the aircraft that appeared on the screen with the goal of guiding them to a safe landing by adjusting their heading, altitude, and speed. When the task began, three aircraft appeared on the display at a height of 4000 ft. and a speed of 250 kts. Two of these aircraft were flying east, parallel to but separately from one another on the north and south sides of the glide path; the other aircraft was flying north, perpendicular to the extension of the glide path. The target airport was shown at the middle of the left edge of the screen. Over time, additional aircraft were scheduled to approach randomly from the north, south, and west. The participants were asked to click on the aircraft and adjust their heading, altitude, and speed separately (namely, when an aircraft approaches the airport, the speed should be adjusted to between 160 kts and 200 kts) in a dialog box positioned at the upper-left corner of the screen with the goal of guiding them to a safe landing. During this process, the participants should maintain safe distances among the aircraft in flight. If the distance between two aircraft at the same height is less than 3 miles, a conflict warning was provided. The participants were required to avoid potential conflicts by changing the height, heading, or speed of the aircraft. The primary task was completed after the participants successfully guided five aircraft to a safe landing.

Given that air traffic controllers are frequently interrupted by air–ground communications (e.g., when a pilot calls for a diversion due to a thunderstorm) while simultaneously monitoring radar screens with the goal of identifying potential conflicts among aircraft, a telephone-answering task was employed as the interruption task. Specifically, since the precise timing of air–ground communications is often unpredictable, the participants in the current experiment were preinformed that they would be required to answer a phone call, but the specific timing of the call was not indicated. In fact, the phone call was made by a research assistant who was blinded to our hypotheses; this call asked the participants to deliver a message to the experimenter verbally and immediately to indicate that it was necessary to change the schedule of the experiment for the following week due to a time conflict. The entire task lasted approximately 15 seconds.

According to Endsley, situation awareness can be assessed via both objective measures, such as the situation awareness global assessment technique (SAGAT), and subjective measures, such as the situation awareness rating technique (SART) [46]. While the SAGAT addresses limitations pertaining to subjective perceptions, it also involves intrusive freezes in task simulations used to collect data, which may disrupt participants’ completion of their tasks. Furthermore, research has indicated that the probe questions in the SAGAT may not accurately assess controllers’ situation awareness because air traffic controllers have a more holistic understanding of situation awareness that cannot be divided into single probing questions pertaining to specific events [47]. In contrast, the SART is a commonly used noninvasive measure of situation awareness that does not interfere with simulated tasks. Therefore, in this study, situation awareness was evaluated via the SART. The SART requires operators to rate their own situation awareness on a continuous 100 mm rating scale for each of three dimensions: demand on attentional resources, supply of attentional resources and understanding of the situation. According to Taylor, the situation awareness score (SA) of each participant can be calculated via the following formula: SA = understanding of the situation–(demand on attentional resources–supply of attentional resources) [48]. The higher a participant’s SA is, the higher his or her level of situation awareness.

### Procedure

Prior to the start of the experiment, the 44 participants were randomly assigned to one of two conditions, i.e., with or without interruption; 22 participants were included in each group (the experimental group vs. the control group). All participants were given standardized instructions and allotted five minutes to familiarize themselves with the simulated air traffic control task. Then, they were asked to complete an assessment of their familiarity with the experimental tasks, the level of simulation, and the adequacy of the practice, which were scored on a 10-point scale. At the beginning of the experiment, each participant was fully engaged in the primary task—i.e., the simulated air traffic control task. They were asked to guide the aircraft shown on the screen to a safe landing while simultaneously monitoring and resolving potential aircraft conflicts. This goal could be achieved by adjusting the heading, altitude, and speed of the aircraft via a mouse.

In the interruption condition, as the third aircraft entered the glide path, the ongoing task was interrupted by a phone call to the laboratory. The participants were asked to answer the phone and communicate the message to the experimenter verbally as previously requested. After approximately 15 s, the participants completed the telephone-answering task and continued to perform the primary task until the fifth aircraft landed successfully. In the no interruption condition, participants performed the simulated air traffic control task continuously until the fifth aircraft landed successfully. While they completed this task, the phone in the experimental room rang. The participants were required to answer the phone call and deliver the message verbally to the experimenter in the same manner as the participants in the interruption condition. After the conclusion of the experimental tasks, all the participants were asked to complete the SART scale anonymously.

### Results

**Fig 1:**
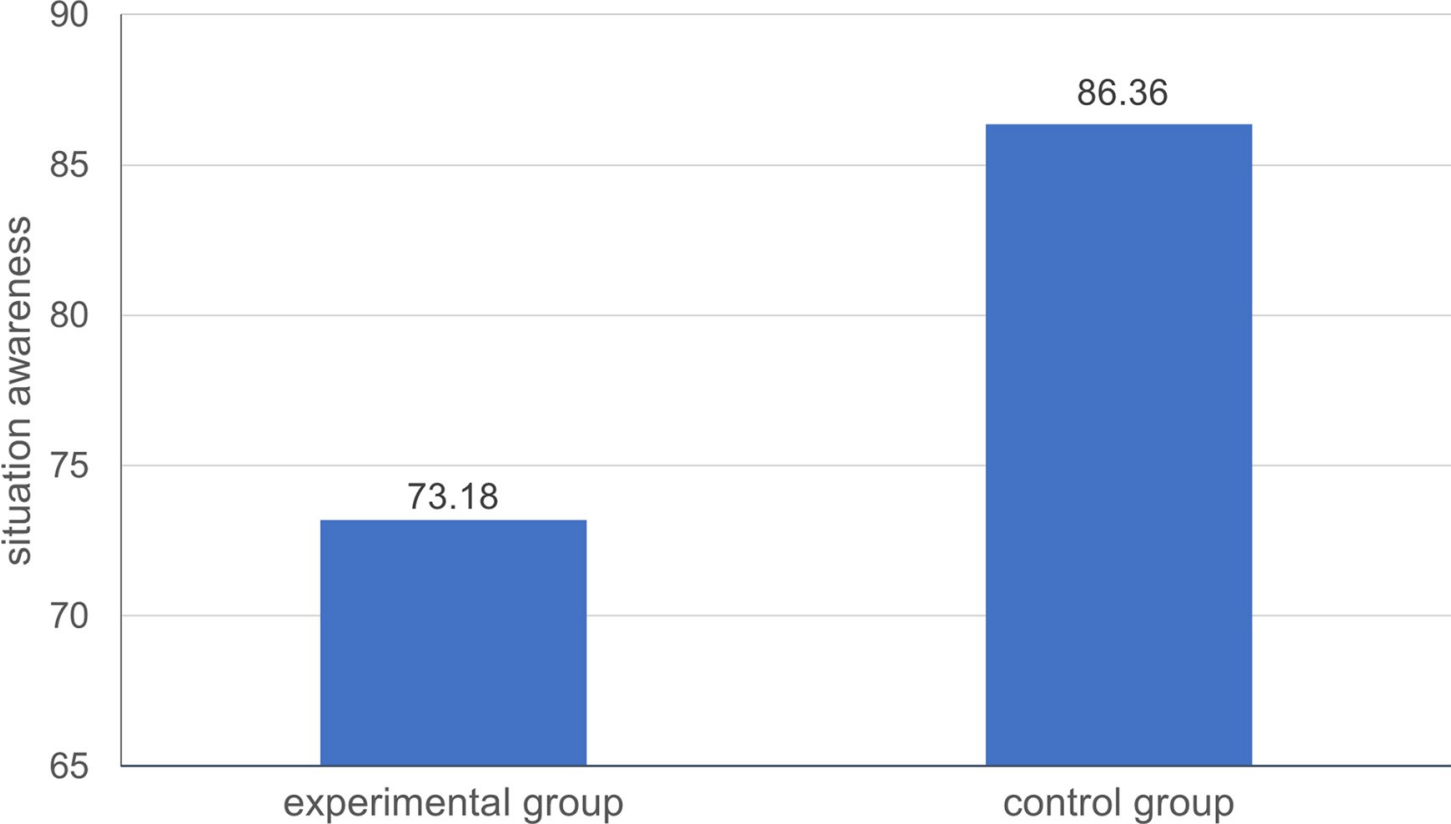
Situation awareness of participants in the two groups.

The SA of each participant was calculated via the formula proposed by Taylor, i.e., SA = understanding of the situation–(demand on attentional resources–supply of attentional resources) [48]. On this basis, an independent sample t test was conducted to determine whether task interruption had a significant effect on the situation awareness of the controllers. The results revealed that the SAs of the participants differed significantly between the interruption condition and the no interruption condition, *t*_(24.33)_ *=* -3.35, *p* < 0.01, Cohen’s *d* = -1.01. The situation awareness of the participants in the experimental group with task interruption (*M*_Experimental group_ = 73.18, *SD*_Experimental group_ = 5.01) was significantly worse than that of the participants in the control group without task interruption (*M*_Control group_ = 86.36, *SD*_Control group_ = 17.74) (Fig 1). These results suggest that interruptions that occur during the process of task execution have a significant negative effect on situation awareness among controllers. When the simulated air traffic control task was interrupted by a secondary task, the level of situation awareness of the controllers decreased significantly.

## Experiment 2

In Experiment 1, we demonstrated that task interruption has a negative effect on situation awareness among air traffic controllers. As previously indicated, the interruptions that occur during daily air traffic control operations often feature different modalities. Specifically, these task interruptions often involve two sensory modalities: visual and auditory. The purpose of Experiment 2 was to investigate whether cross-modal interruptions have a less disruptive impact on controllers’ situation awareness than do intramodal interruptions as well as whether a visual interruption of a visual primary task could represent a greater threat to controllers’ situation awareness than do other forms of interruption.

### Participants

We used G*Power (3.19.7) software to conduct a priori sample size estimation, in which context the statistical power was set at 0.8 and the threshold for a large effect size was set at .04 for F tests; the alpha level was set at .01, and the results revealed that at least 77 participants were required to detect a significant effect with 80% power. Ultimately, we recruited a sample of 80 healthy male volunteers, whose average age was twenty-two years. The entry criteria used for this experiment were the same as those employed in Experiment 1. All these participants were new graduates majoring in air traffic control who had previously received radar control simulation training for the same amount of time and understood the interval standard for aircraft flight. Before the beginning of the experiment, the participants were advised of the anonymity of the results. Oral informed consent was obtained from each participant. After the participants completed the experiment, they were thanked and debriefed.

### Task and materials

To investigate how task modality moderates the impact of task interruption on situation awareness, two different primary task modalities (visual, auditory) and two different interruption modalities (visual, auditory) were considered in the present experiment. Consequently, four tasks were employed, including a visual primary task, an auditory primary task, a visual interruption task, and an auditory interruption task.

The auditory primary task employed in this study was a simulated air‒ground communication task. Each participant was asked to engage in simulated air‒ground communication with a student pilot (P) according to two nonroutine air‒ground communication scenarios (in Scenario 1, the pilot requested to yaw to avoid bad weather, while in Scenario 2, the pilot requested to land early due to a lack of fuel). Each participant was assumed to be an air traffic controller at Tianjin international airport. An "approach plate" that depicted an instrument approach procedure for an instrument landing system (ILS) approach to the airport was provided to the participants. The visual interruption task that we used was an image identification task. The participant was presented with an ambiguous figure in which a tree was depicted such that the lines of the branches appeared to include many hidden heads. The participants were asked to mark the positions of at least nine heads within 15 seconds. The visual primary task was the simulated air traffic control task, while the auditory interruption task was the telephone-answering task. Both of these two tasks were the same as those used in Experiment 1.

### Procedure

Two different primary task modalities (visual, auditory) and two different interruption modalities (visual, auditory) were manipulated across four different conditions: the auditory–visual condition, the auditory–auditory condition, the visual–auditory condition, and the visual–visual condition. In the auditory–visual condition, the auditory interruption task was inserted into the visual primary task. In the auditory–auditory condition, the auditory interruption task was inserted into the auditory primary task. In the visual–auditory condition, the visual interruption task was inserted into the auditory primary task. In the visual–visual condition, visual interruption occurred during the visual primary task. Each of the participants was randomly assigned to one of those four experimental conditions; 20 participants were included in each condition.

After the experimenter introduced the experimental tasks to the participants, they were given five minutes to familiarize themselves with their primary tasks. Then, they were asked to complete an assessment of their familiarity with those tasks, the level of simulation, and the adequacy of the practice on a 10-point scale. At the beginning of the experiment, each participant was fully engaged in their primary task. In the visual–visual condition and the auditory–visual condition, the participants were first required to perform the simulated air traffic control task on their own. As the third aircraft entered the glide path, the simulated air traffic control task was interrupted by the insertion of an interruption task. Specifically, participants in the visual–visual condition were asked to mark the positions of at least nine heads that were hidden in an ambiguous figure within 15 seconds, whereas participants in the auditory–visual condition were required to take approximately 15 seconds to answer a phone call and verbally communicate the corresponding message to the experimenter in accordance with the requirements. After the interruption task was completed, the participants were asked to resume the simulated air traffic control task until the fifth aircraft landed successfully. In the auditory–auditory and visual–auditory conditions, the participants were first required to perform the simulated air–ground communication task. When the communication task reached the second scenario, it was interrupted by the insertion of an interruption task. Specifically, in the auditory–auditory condition, this task was interrupted by the telephone-answering task. In contrast, in the visual–auditory condition, the task was interrupted by the image identification task. After the interruption task was completed, the participants were required to resume the simulated air–ground communication task until the complete communication process had been concluded. After the experimental tasks ended, the participants in all the conditions completed the SART scale anonymously.

### Results

When the four experimental conditions were compared, the situation awareness of the participants was revealed to be significantly different, *F*_(3,76)_ = 20.136, *p* < 0.001, η^2^ = 0.443. The situation awareness of the participants in the visual–visual condition was the worst among all participants (*M*_visual-visual_ = 50.25, *SD*_visual-visual_ = 10.70), whereas the situation awareness of participants in the visual–auditory-condition was much better (_Mvisual-auditory_ = 74.5, _SDvisual-auditory_ = 13.17). The order of SAs among participants in the four experimental conditions was as follows: *M*_visual-visual_ <*M*_auditory-auditory_ <*M*_auditory-visual_ <*M*_visual-auditory_. These results indicated that the interruption of an auditory primary task by a visual secondary task was the least disruptive with respect to situation awareness. However, the interruption of a visual primary task by a visual secondary task was the most disruptive.

**Fig 2:**
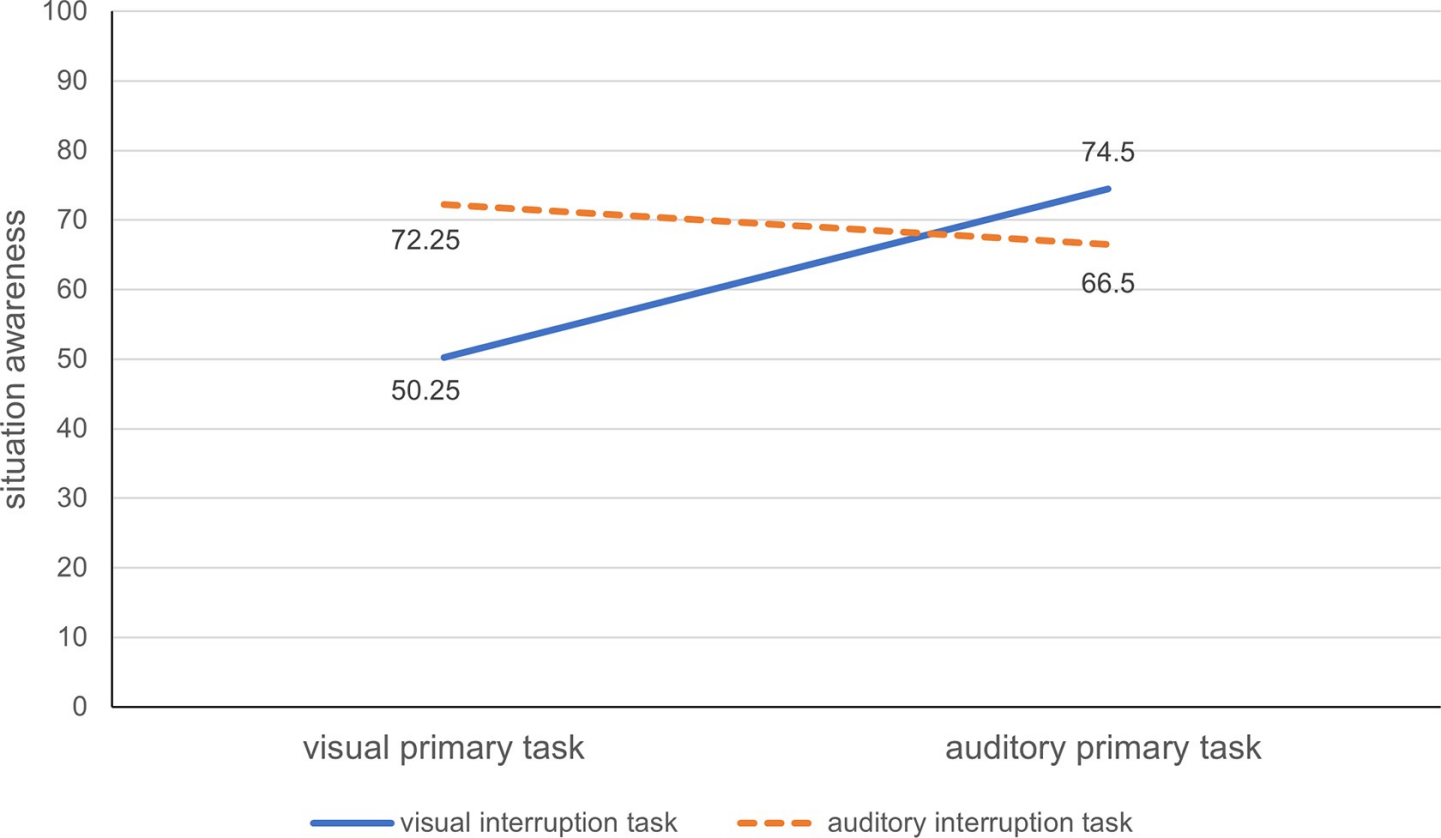
Situation awareness of participants under the four experimental conditions.

The effects of primary task modality and interruption modality on situation awareness were examined by conducting a 2×2 two-way analysis of variance (ANOVA). A significant interaction was observed between the primary task modality and the interruption modality, *F*_(1,76)_ = 37.8, *p* < 0.001, η^2^ = 0.332. A simple effect analysis conducted to decompose the interaction indicated that the incorporation of a visual interruption into a visual primary task (i.e., the simulated air traffic control task) resulted in significantly lower situation awareness than did the incorporation of an auditory interruption, *t*_38_ = -6.869, *p* < 0.001. Similarly, the incorporation of an auditory interruption into an auditory primary task was also found to be more disruptive with respect to participants’ situation awareness than was the incorporation of a visual interruption, *t*_38_ = 2.173, *p* < 0.05 (Fig 2). These results consistently indicated that the effects of intramodal interruptions (visual–visual and auditory–auditory) on situation awareness were more disruptive than those of cross-modal interruptions (auditory–visual and visual–auditory).

Notably, the main effects of interruption modality (*F*_(1, 76)_ = 8.232, *p* < 0.01) and primary task modality (*F*_(1, 76)_ = 14.375, *p* < 0.001) were both statistically significant. Thus, a visual interruption led to significantly lower levels of situation awareness than did an auditory interruption (*M*_visual interruption_ = 62.38, *M*_auditory interruption_ = 69.38). Additionally, the reduction observed in the situation awareness of the operator was greater when the visual primary task was interrupted than when the auditory primary task was interrupted (*M*_visual interrupted_ = 61.25, *M*_auditory interrupted_ = 70.5). The raw data supporting these findings can be found in (see S1 File).

## General discussion

The results of this study confirmed that interruptions that occur during an air traffic control task have negative effects on the situation awareness of operators. When the simulated air traffic control task in this research was interrupted by the insertion of a secondary task (e.g., the telephone-answering task), the situation awareness of controllers was significantly reduced. These findings can probably be explained by reference to the attentional allocation model, which suggests that individuals have limited attentional resources that they can use to process given information. According to the attentional allocation model [49], when sufficient resources are allocated to accomplish both tasks in parallel, dual tasks can arise. However, this situation typically occurs when one of the tasks in question is automated and requires minimal attentive resources, such as walking over a small puddle while conversing with a friend [50]. In most cases, it is challenging to achieve genuine simultaneity in the context of multitasking. Numerous studies have reported that our cognitive abilities are severely limited when we perform several tasks simultaneously or in close succession [51,52]. Thus, people are more likely to employ the strategy of task switching in response to multiple tasks, especially when those tasks are complex or unfamiliar and require the allocation of resources to working memory [50].

In the context of the simulated air traffic control task included in Experiment 1, which involves complex and dynamically changing information, participants are responsible for prioritizing approaching aircraft, guiding them to land sequentially, and concurrently monitoring and resolving flight conflicts under the influence of time pressure. During the interruption task, participants are required to answer a phone call and deliver a message verbally to the experimenter as an immediate response. The complexity of both tasks requires participants to retain crucial information from the simulated air traffic control task in their working memory, thus enabling them to resume that task after completing the telephone-answering task. This interruption leads to a failure on the part of participants to allocate the necessary resources effectively to the simulated air traffic control task to process information in a timely and continuous manner. Ultimately, this situation inevitably results in a relatively poor level of situation awareness.

Furthermore, the results of Experiment 2 reveal that the effect of task interruption on situation awareness is moderated by task modality. More specifically, the effect of intramodal interruptions on situation awareness is revealed to be more disruptive than that of cross-modal interruptions. when the visual primary task follows a visual interruption task, the resulting decrease in situation awareness is greater than when it follows an auditory interruption task. These findings are compatible with multiple resource theory [43,44], which suggests that different sensory channels (such as vision, hearing, or touch) are associated with separate attentional resources. The concurrent performance of two tasks can be improved if these tasks are presented via different sensory modalities. This situation may explain why, in Experiment 2, the decrease observed in situation awareness among the controllers was greater when the simulated air–ground communication was disrupted by the telephone-answering task, which also placed demands on the auditory channel, than by image identification, which involved visual input. In addition, some recent studies in the field of cognitive neuroscience may have provided insights that can enable us to obtain a better understanding of our findings. For example, findings that have been reported in the research on working memory and mental imagery have indicated that activity occurs in multimodal cortical regions that are involved in sustaining the current representation in a complex, multimodal form rather than suppressing competing stimuli [53,54].

More interestingly, the results of our study also reveal that visual interruption is associated with more substantial decreases in situation awareness than is auditory interruption; furthermore, interruption of the visual task entails greater decreases in situation awareness than does interruption of the auditory task. Altmann and Trafton’s memory for goals theory suggests that maintaining an association between the suspended primary task goal and relevant environmental cues is critical to the task resumption process [42,55,56]. The auditory interruption facilitates resumption to the degree that the interruption in question allows the environmental cues and the association with the suspended primary task goal to be maintained [42]. In contrast, both visual interruption and interruption of a visual task make it more difficult for operators to pay close attention to the relevant environmental context, thus increasing the difficulty of maintaining a high level of situation awareness in this context.

Although the experiments reported here reveal consistent and robust effects, the results of our study should be viewed as exploratory. Future research can be expected to make several improvements in this regard. First, the results of our study are limited due to our reliance on simulated tasks, which may not be able to represent the real situation precisely. The generalizability of our findings must be demonstrated by reference to real-world scenarios featuring high ecological validity in future studies. Second, future research on the effects of multiple interruptions may offer more comprehensive insights into the ways in which interruption frequency affects situation awareness. Third, future research should consider incorporating more objective measurements, such as eye movement and electroencephalogram (EEG) methodologies, to overcome the limitations entailed by the fact that the SART measures subjective perceptions of situation awareness. Additionally, the situation awareness exhibited by air traffic controllers can apparently be affected by their cognitive ability, working memory capacity, and familiarity with the tasks at hand as well as the relevance among those tasks and other factors. Thus, future studies can measure these factors to shed additional light on the roles played by these important factors in moderating the effects of interruptions to air traffic control tasks on situation awareness. Finally, the precise relationship between situation awareness and performance remains unclear [57]. Poor performance may be caused by many factors: when the notion of SA is incomplete or inaccurate, when the correct action in the identified situation is not known or calculated, or when time or other factors limit a person’s ability to perform the correct action [58]. Therefore, future research should consider investigating how variables such as personal experience, task complexity, time pressure, and workload interact with situation awareness to influence task performance. Such research can provide a more comprehensive understanding of the interactions among interruptions, situation awareness, and task performance, thereby offering valuable guidance for the future of the complex field of air traffic management.

## Conclusion and recommendations

Our findings generally indicate that interruptions in a simulated air traffic control task led to decreased situation awareness on the part of controllers. Intramodal interruptions are revealed to be more disruptive with regard to situation awareness than are cross-modal interruptions. For example, when air traffic control operations are disrupted by the requirement to identify various ambiguous images simultaneously via the visual channel, the decrease observed in the situation awareness of air traffic controllers is greater than that by tasks involving answering a phone call, which pertain to the auditory channel. We hope that our findings can help improve the design of human-computer interaction in an automation system for air traffic control by improving our understanding of the effects of interruptions on situation awareness. Multiple intersecting aviation situational tasks often cause air traffic controllers to face issues pertaining to interruption and task switching. In the context of typical air traffic control operations, the controllers must address competing attentional demands and perform multiple concurrent tasks, which can involve interruptions by other human or machine agents. The findings of this study suggest that if an interruption cannot be avoided, it could be beneficial to design systems that are resistant to such interruptions and that can help human operators maintain situation awareness. Perhaps offering controllers the option to choose between visually or audibly marking significant states, such as impending aircraft conflicts, before a mission interruption is a potential approach worth exploring, as this flexibility allows controllers to select sensory modalities distinct from their tasks, thereby aiding in the maintenance of situational awareness. However, it should be acknowledged that this proposal is preliminary and necessitates further empirical investigation to fully establish its effectiveness.

## Supporting information

S1 File The data set. The raw data supporting the findings described in manuscript.
(XLSX)
